# Development and validation of a hyperlipidemia risk prediction model for middle-aged and older adult Chinese using 2015 CHARLS data

**DOI:** 10.3389/fpubh.2025.1420596

**Published:** 2025-01-21

**Authors:** Li-xiang Zhang, Shan-Bing Hou, Fang-fang Zhao, Ting-ting Wang, Ying Jiang, Xiao-juan Zhou, Jiao-yu Cao

**Affiliations:** ^1^Division of Life Science and Medicine, Department of Cardiology, The First Affiliated Hospital of USTC, University of Science and Technology of China, Hefei, China; ^2^Division of Life Science and Medicine, Department of Emergency, The First Affiliated Hospital of USTC, University of Science and Technology of China, Hefei, China; ^3^Division of Life Science and Medicine, Department of Rehabilitation Medicine, The First Affiliated Hospital of USTC, University of Science and Technology of China, Hefei, China

**Keywords:** Chinese, middle-aged and older adult individuals, hyperlipidemia, risk factors, nomogram, prediction model

## Abstract

**Objective:**

To develop and validate a predictive model for hyperlipidemia risk among middle-aged and older adult individuals in China, this study aims to offer an effective screening tool for identifying those at risk.

**Methods:**

In this study, we included 6,629 middle-aged and older adult individuals, aged 45 and above, who met the inclusion criteria from the 2015 China Health and Retirement Longitudinal Study (CHARLS) as our research subjects. Utilizing the LASSO regression and multivariate Logistic regression method, we analyzed the independent risk factors associated with hyperlipidemia among these subjects. Subsequently, we established a risk prediction model for hyperlipidemia in the middle-aged and older adult population using statistical software Stata 17.0.

**Results:**

The prevalence rate of hyperlipidemia among the 6,629 middle-aged and older adult participants was 26.32% (1,745 out of 6,629). The LASSO regression and multivariate Logistic regression analysis all revealed that Body Mass Index (BMI), fasting blood glucose, serum uric acid, C-reactive protein, and white blood cell count were independent risk factors for hyperlipidemia in this demographic (with Odds Ratios (OR) greater than 1 and *p*-values less than 0.05). From these findings, a nomogram prediction model was constructed to estimate the risk of hyperlipidemia for middle-aged and older adult individuals. The Area Under the Receiver Operating Characteristic (ROC) Curve (AUC) for the nomograms was 0.717 (95% Confidence Interval (CI): 0.703–0.731), indicating good discrimination. The Decision Curve Analysis (DCA) demonstrated that when the probability of hyperlipidemia in the middle-aged and older adult population falls between 0.11 and 0.61, the application of the nomogram yields the highest net benefit, suggesting that the nomogram model possesses good clinical applicability. The Spiegelhalter’s z-statistic test confirmed that the predicted probabilities from the nomogram model are in good agreement with the observed frequencies of hyperlipidemia (*p* = 0.560). The Brier score for the nomogram model was 17.1%, which is below the threshold of 25%, indicating good calibration. To internally validate the nomogram model, we performed bootstrap resampling 500 times. The C-statistic for the nomogram model from this internal validation was 0.716, and the Brier score was 11.4%, suggesting that the model not only has good predictive efficiency but also good stability.

**Conclusion:**

The nomogram model, which incorporates the identified risk factors for hyperlipidemia in middle-aged and older adult individuals, has demonstrated good predictive efficiency and clinical applicability. It can serve as a valuable tool to assist healthcare professionals in screening for high-risk groups and implementing targeted preventive interventions. By doing so, it has the potential to significantly reduce the incidence of hyperlipidemia among this demographic.

## 1 Introduction

Hyperlipidemia, a component of dyslipidemia, is characterized by elevated levels of one or more plasma lipids resulting from abnormalities in fat metabolism. This metabolic disorder can precipitate atherosclerosis, which underlies the pathology of numerous diseases and stands as a crucial risk factor for both coronary atherosclerotic heart disease and cerebrovascular disease (1). Hyperlipidemia is a significant comorbidity in cardiovascular health, with over 50% of ischemic cardiomyopathy cases being linked to this metabolic disorder. Moreover, the presence of hyperlipidemia is associated with an increased risk of mortality from cardiovascular diseases (2, 3). Owing to the deterioration of physiological functions associated with aging, the prevalence of hyperlipidemia among middle-aged and older adult individuals is observed to rise annually (4). Hyperlipidemia encompasses several subtypes, including hypertriglyceridemia, hypercholesterolemia, and mixed hyperlipidemia (5). A research report published by Nature journal in 2020 assessed data from 200 countries and revealed that in 1980 (6), the average levels of Total Cholesterol (TC) and non-high-density lipoprotein cholesterol (HDL-C) in China were among the lower grades globally, significantly lower when compared to levels in western countries. However, by 2018, the average levels of TC and non-HDL-C among adults in China had reached or even surpassed the averages observed in some of these western nations. This shift indicates a potential trend of increasing cardiovascular disease risk for Chinese residents, which parallels the rising prevalence of hyperlipidemia. Given the close relationship between hyperlipidemia and cardiovascular and cerebrovascular diseases, it is crucial to assess the high-risk factors associated with hyperlipidemia, predict its risk, and implement appropriate preventive measures. This proactive approach can significantly contribute to the prevention of cardiovascular and cerebrovascular diseases (7). Furthermore, hyperlipidemia’s pathological mechanism stems from abnormal blood lipid levels, and given that it is a controllable condition, the importance of early detection and proactive intervention cannot be overstated. Timely identification of the condition and the initiation of appropriate medical and lifestyle interventions are paramount in managing this disease effectively (8). Currently, there is a scarcity of representative studies focusing on the risk prediction models for hyperlipidemia specifically in the middle-aged and older adult demographic, both domestically and internationally. Addressing this gap, the present study develops a hyperlipidemia risk prediction model tailored for this age group using survey data from the China Health and Retirement Longitudinal Study (CHARLS). The goal is to provide a reference that can inform the prevention and management strategies for hyperlipidemia in middle-aged and older adult populations.

## 2 Methods

### 2.1 Research objects

**Figure 1:**
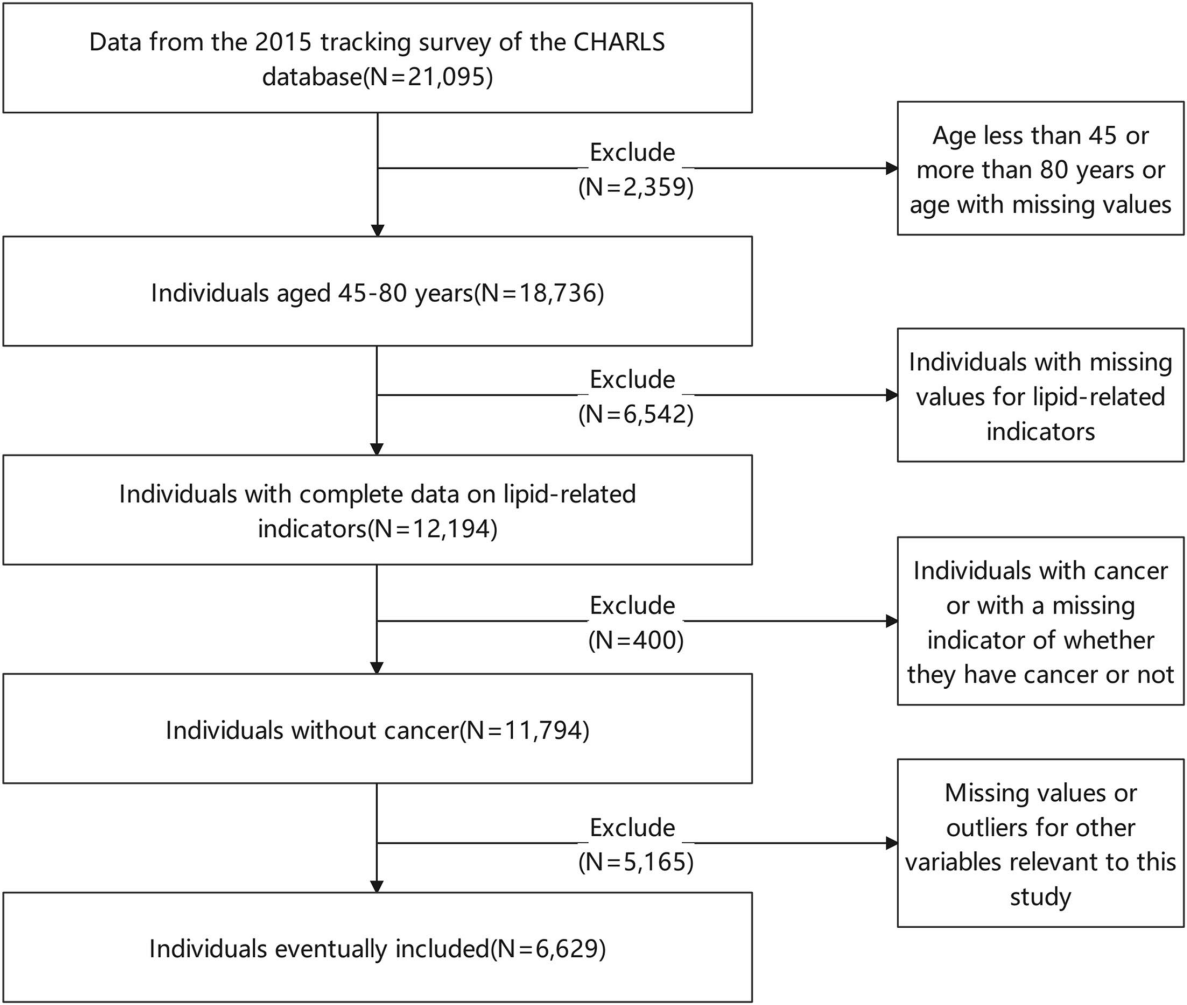
Flow chart of screening research objects.

For the purposes of this study, we utilized the 2015 China Health and Retirement Longitudinal Study (CHARLS) survey data. This data was selected by the researcher and used with appropriate permissions. The CHARLS is a longitudinal survey specifically designed to study individuals aged 45 and above in China, and it is administered by the National Development Research Institute of Peking University. The national baseline survey for CHARLS was initiated in 2011, with residents aged 45 or above being randomly selected from 28 provincial administrative units, 150 county-level units, and 450 village-level units for follow-up assessments every 2 to 3 years. The CHARLS database is a rich resource that includes high-quality, detailed microdata on various aspects of the middle-aged and older adult population, such as basic demographic information; health status and functionality; healthcare utilization and insurance coverage; employment, retirement, and pension details; income, expenditure, and asset information. This data set is valuable as it provides a comprehensive view of the health status of China’s middle-aged and older adult population (9, 10). The inclusion criteria for participant selection in this study were as follows: (1) baseline age ranging from 45 to 80 years old; (2) Completeness of blood test results, past medical history of chronic diseases, demographics, and measurement indicators. The exclusion criteria included: (1) Outliers in the research index data; (2) Missing values within the research index data; (3) Individuals with a history of cancer. After applying these criteria, a total of 6,629 individuals were enrolled in the study. For a detailed depiction of the screening process, refer to Figure 1. The China Health and Retirement Longitudinal Study (CHARLS) has received approval from the Biomedical Ethics Review Committee of Peking University (with the approval number IRB00001052-11015). This ensures that the study adheres to ethical standards and protocols. Furthermore, all respondents have provided their informed consent to participate in the investigation, safeguarding their rights and contributing to the study’s legitimacy (11, 12).

### 2.2 Research related indicators

The research encompasses three primary components: questionnaire interviews, physical examinations, and laboratory tests. Questionnaire interviews are conducted by trained investigators who collect information on the interviewee’s gender, age, marital status, and history of smoking and drinking, as well as any past chronic diseases, including hypertension, diabetes, chronic lung disease, liver disease, heart disease, stroke, kidney disease, gastrointestinal diseases, emotional and mental disorders, memory-related conditions, arthritis, and asthma. Physical measurements such as the interviewee’s height and weight are taken by healthcare professionals. For laboratory analysis, fasting venous blood samples are collected from interviewees who have fasted for at least 12 h by medical staff. A range of biochemical parameters are then assessed, including fasting blood glucose (FBG), glycated hemoglobin (HbA1c), triglyceride (TG), total cholesterol (TC), high-density lipoprotein cholesterol (HDL-C), low-density lipoprotein cholesterol (LDL-C), uric acid (UA), creatinine, red blood cell volume, hemoglobin, white blood cell count, and serum creatinine.

### 2.3 Categories and definitions of indicators

The gender categories are binary, including only “male” and “female.” Marital status is categorized into “unmarried/divorced/widowed” and “have a spouse.” Smoking history is simplified into a binary choice of “yes” or “no.” Drinking history is categorized based on frequency: “more than once a month,” “once a month or less,” and “no drinking.” Past medical history regarding chronic conditions such as hypertension, diabetes, chronic lung disease, liver disease, heart disease, stroke, kidney disease, gastrointestinal diseases, emotional and mental diseases, memory-related diseases, arthritis, and asthma is also dichotomized into “yes” or “no” categories. Specifically, diabetes is diagnosed if any of the following criteria are met (13): Fasting blood glucose is greater than or equal to 7.0 mmol/L, The glucose tolerance test result after 2 h is greater than or equal to 11.1 mmol/L, Glycosylated hemoglobin is greater than or equal to 6.5%. Additionally, individuals with a previous diagnosis of diabetes or those who are undergoing hypoglycemic therapy are also classified under the diabetes category. Hypertension is diagnosed when an individual presents with a systolic blood pressure (SBP) of 140 mmHg or higher and/or a diastolic blood pressure (DBP) of 90 mmHg or higher, according to standard clinical criteria. Additionally, individuals who are currently undergoing treatment with antihypertensive medications are also classified as having hypertension (14). The Body Mass Index (BMI) is calculated using the formula: weight in kilograms (kg) divided by the square of height in meters (m^2) (13). Heart disease encompasses a spectrum of conditions that affect the heart’s function and structure. This includes myocardial infarction, which is commonly known as a heart attack; coronary heart disease, often referred to as CHD and related to the narrowing of the coronary arteries; angina pectoris, a type of chest pain resulting from insufficient oxygen supply to the heart; congestive heart failure, where the heart is unable to pump blood effectively; and other related heart diseases (11). A subject can be diagnosed with hyperlipidemia if the diagnostic results meet at least one of the following criteria: Triglyceride (TG) levels are greater than or equal to 2.3 mmol/L, Total Cholesterol (TC) levels are greater than or equal to 6.2 mmol/L, Low-Density Lipoprotein Cholesterol (LDL-C) levels are greater than or equal to 4.1 mmol/L, High-Density Lipoprotein Cholesterol (HDL-C) levels are less than 1.0 mmol/L (15).

### 2.4 Statistical analysis

For data analysis, Stata 17.0 was employed. For measurement data that followed a skewed distribution, the summary statistics are given as Median (Q1, Q3), and the Mann–Whitney U test was utilized for group comparisons. Categorical data are described in terms of frequency and percentage, and the Pearson chi-square test was applied for group comparisons. The Least Absolute Shrinkage and Selection Operator (LASSO) regression analysis was conducted to identify the independent risk factors for hyperlipidemia among middle-aged and older adult individuals. Based on these identified factors, a nomogram prediction model was developed by using the Nomolog software programs in Stata 17.0 (16). The discrimination ability of the nomogram was evaluated using the Area Under the Receiver Operating Characteristic (ROC) Curve. The clinical applicability was assessed with the Decision Curve Analysis (DCA). The calibration of the nomogram model was evaluated using the Hosmer-Lemeshow goodness of fit test, Brier score, and calibration curve. To prevent over-fitting, the Bootstrap resampling method was implemented for internal validation of the nomogram. Bilateral tests were conducted, and results were considered statistically significant when the *p*-value was less than 0.05.

## 3 Results

### 3.1 Comparison of data between hyperlipidemia group and non-hyperlipidemia group

The study sample comprised 6,629 middle-aged and older adult individuals with a median age of 58.00 years, with interquartile range (IQR) of (51.00, 64.00) years. The gender distribution was as follows: 3,161 males (47.68%) and 3,468 females (52.32%). For a comprehensive overview of other demographic and health-related survey data, refer to Table 1. Within this population, hyperlipidemia was identified in 26.32% (1,745 out of 6,629) of the individuals. A comparative analysis between the hyperlipidemia group and the non-hyperlipidemia group revealed statistically significant differences in several health metrics and conditions, including Age, BMI, fasting blood glucose, blood urea nitrogen, uric acid, glycosylated hemoglobin, hemoglobin, white blood cell count, mean red blood cell volume, Platelet count, C-reactive protein, hypertension, diabetes, and gender (*p* < 0.05). Detailed data comparing these groups can be found in Table 1.

**Table 1:** Comparison of data between hyperlipidemia group and non-hyperlipidemia group.

Variables	Total (*n* = 6,629)	Non-hyperlipidemia group (*n* = 4,884)	Hyperlipidemia group (*n* = 1745)	Statistic	*p*-value
Age (years), M (Q₁, Q₃)	58.00 (51.00, 64.00)	58.00 (51.00, 65.00)	57.00 (51.00, 64.00)	Z = −2.06	**0.039**
BMI(kg/m^2), M (Q₁, Q₃)	23.27 (21.14, 25.71)	22.86 (20.79, 25.18)	24.60 (22.27, 26.99)	Z = −16.85	**<0.001**
Fasting blood glucose (mg/dl), M (Q₁, Q₃)	93.69 (86.49, 102.70)	93.69 (86.49, 100.90)	97.30 (90.09, 109.91)	Z = −15.18	**<0.001**
Blood urea nitrogen (mg/dl), M (Q₁, Q₃)	14.57 (12.32, 17.65)	14.85 (12.32, 17.93)	14.29 (12.04, 17.09)	Z = −4.77	**<0.001**
Serum uric acid (mg/dl), M (Q₁, Q₃)	4.70 (3.90, 5.60)	4.50 (3.80, 5.40)	5.10 (4.30, 6.10)	Z = −14.98	**<0.001**
Glycosylated hemoglobin (%), M (Q₁, Q₃)	5.70 (5.50, 6.00)	5.70 (5.50, 6.00)	5.80 (5.50, 6.10)	Z = −7.98	**<0.001**
Hemoglobin (mg/dl), M (Q₁, Q₃)	13.60 (12.50, 14.80)	13.50 (12.40, 14.70)	13.90 (12.80, 15.20)	Z = −8.16	**<0.001**
White blood cell count (×10 9/L), M (Q₁, Q₃)	5.65 (4.70, 6.80)	5.50 (4.60, 6.60)	6.00 (4.98, 7.20)	Z = −10.64	**<0.001**
Mean red blood cell volume (FL), M (Q₁, Q₃)	92.00 (88.00, 95.70)	92.10 (88.10, 95.80)	91.60 (87.80, 95.40)	Z = −2.48	**0.013**
Platelet count (×10^9/L), M (Q₁, Q₃)	203.00 (161.00, 245.00)	201.00 (159.00, 243.00)	210.00 (169.00, 250.00)	Z = −4.62	**<0.001**
Serum creatinine (mg/dl), M (Q_1_, Q_3_)	0.75 (0.65, 0.88)	0.75 (0.65, 0.87)	0.76 (0.65, 0.89)	Z = −1.24	0.217
C-reactive protein (mg/l), M (Q_1_, Q_3_)	1.20 (0.70, 2.30)	1.00 (0.60, 1.90)	2.10 (1.20, 3.60)	Z = −25.68	**<0.001**
Drinking, *n* (%)				*χ*^2^ = 0.83	0.661
More than once a month	1949 (29.40)	1,450 (29.69)	499 (28.60)		
Once a month and below	627 (9.46)	457 (9.36)	170 (9.74)		
No drinking	4,053 (61.14)	2,977 (60.95)	1,076 (61.66)		
Hypertension, *n* (%)				*χ*^2^ = 13.46	**<0.001**
No	6,069 (91.55)	4,508 (92.30)	1,561 (89.46)		
Yes	560 (8.45)	376 (7.70)	184 (10.54)		
Diabetes, *n* (%)				*χ*^2^ = 20.76	**<0.001**
No	6,490 (97.90)	4,805 (98.38)	1,685 (96.56)		
Yes	139 (2.10)	79 (1.62)	60 (3.44)		
Chronic lung disease, *n* (%)				*χ*^2^ = 0.65	0.420
No	6,516 (98.30)	4,797 (98.22)	1719 (98.51)		
Yes	113 (1.70)	87 (1.78)	26 (1.49)		
Liver disease, *n* (%)				*χ*^2^ = 0.48	0.487
No	6,566 (99.05)	4,840 (99.10)	1726 (98.91)		
Yes	63 (0.95)	44 (0.90)	19 (1.09)		
Heart disease, *n* (%)				*χ*^2^ = 2.41	0.121
No	6,447 (97.25)	4,759 (97.44)	1,688 (96.73)		
Yes	182 (2.75)	125 (2.56)	57 (3.27)		
Stroke, *n* (%)				*χ*^2^ = 0.14	0.711
No	6,595 (99.49)	4,858 (99.47)	1737 (99.54)		
Yes	34 (0.51)	26 (0.53)	8 (0.46)		
Emotional and mental diseases, *n* (%)				*χ*^2^ = 0.46	0.497
No	6,616 (99.80)	4,876 (99.84)	1740 (99.71)		
Yes	13 (0.20)	8 (0.16)	5 (0.29)		
Memory-related diseases, *n* (%)				*χ*^2^ = 0.64	0.424
No	6,595 (99.49)	4,861 (99.53)	1734 (99.37)		
Yes	34 (0.51)	23 (0.47)	11 (0.63)		
Gender, *n* (%)				*χ*^2^ = 15.24	**<0.001**
Male	3,161 (47.68)	2,259 (46.25)	902 (51.69)		
Female	3,468 (52.32)	2,625 (53.75)	843 (48.31)		
Marital status, *n* (%)				*χ*^2^ = 2.67	0.102
Unmarried/divorced/widowed	691 (10.42)	527 (10.79)	164 (9.40)		
Have a spouse	5,938 (89.58)	4,357 (89.21)	1,581 (90.60)		

Z: Mann–Whitney test, *χ*^2^: Chi-square test, M: Median, Q₁: 1st Quartile, Q₃: 3st Quartile, BMI: body mass index.

Bold values represent statistically significant differences, with *p*-value < 0.05.

### 3.2 Screening of characteristic variables for hyperlipidemia in middle-aged and older adult people

**Figure 2:**
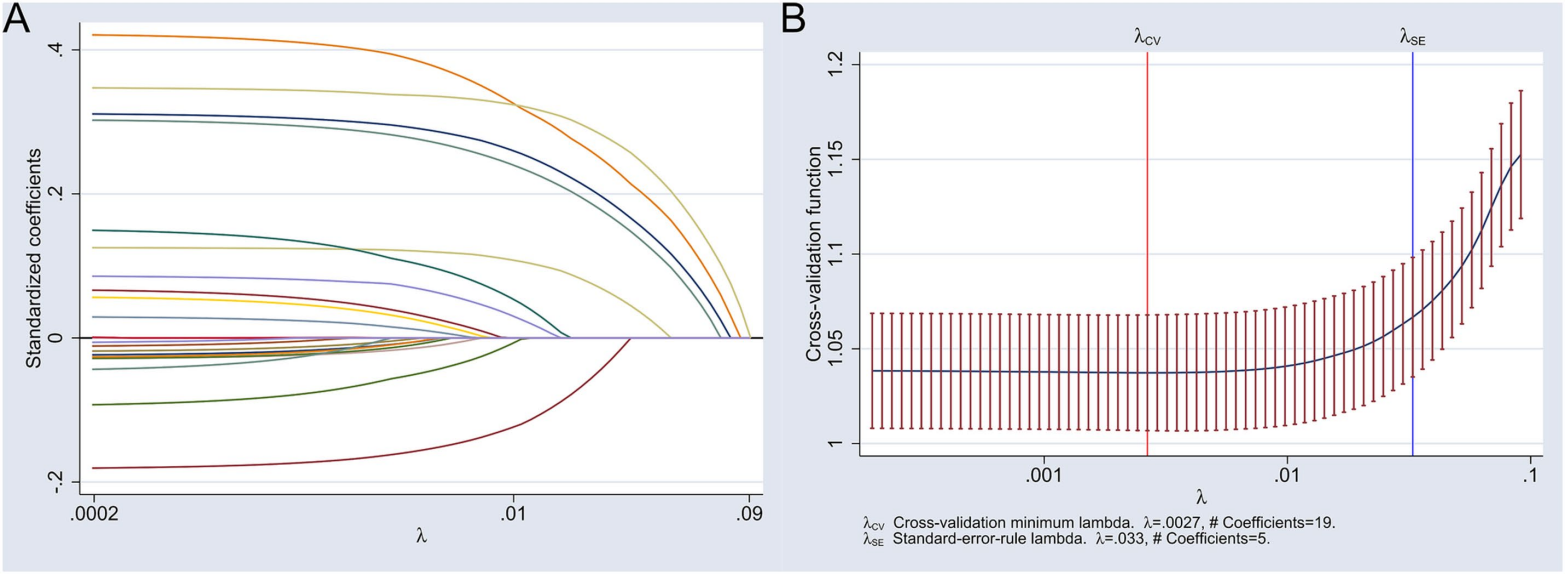
Results of the LASSO analysis for screening characteristic variables of hyperlipidemia in middle-aged and older adult people. **(A)** Variable coefficient path diagram; **(B)** Cross-validation plot with 1 standard error bounds.

The LASSO regression was used to screen characteristic variables for hyperlipidemia in middle-aged and older adult people. The 23 indicators in Table 1 were used as independent variables in the LASSO regression, and the occurrence of hyperlipidemia in middle-aged and older adult people was used as the dependent variable. A 10-fold cross-validation was conducted, with the variables corresponding to the minimum Lambda.1se from the cross-validation selected as the characteristic variables for hyperlipidemia in the middle-aged and older adult. The results showed that the minimum Lambda.1se was 0.033, and at this point, there were a total of 5 corresponding characteristic variables, namely fasting blood glucose, uric acid, C-reactive protein, white blood cell count, and BMI. See Figure 2.

### 3.3 Multivariate logistic regression analysis of hyperlipidemia in middle-aged and older adult individuals

Utilizing the five feature variables selected by LASSO regression as independent variables and the incidence of hyperlipidemia as the dependent variable, a multivariate Logistic regression model was developed. The analysis revealed that BMI, fasting blood glucose, serum uric acid, C-reactive protein, and white blood cell count were significant independent risk factors for hyperlipidemia in the middle-aged and older adult population. The results indicated that for these factors, the odds ratios (OR) were greater than 1 and the *p*-values were less than 0.05, which is indicative of statistical significance. For a detailed examination of these results, please refer to Table 2.

**Table 2:** The results of multivariate logistic regression analysis of hyperlipidemia in middle-aged and older adult people.

Variables	β	S.E	Z	*p*	OR (95%CI)
BMI	0.11	0.01	13.01	<0.001	1.12 (1.10 ~ 1.14)
Fasting blood-glucose	0.01	0.00	9.47	<0.001	1.01 (1.01 ~ 1.01)
Uric acid	0.25	0.02	11.40	<0.001	1.28 (1.23 ~ 1.34)
C-reactive protein	0.07	0.01	8.56	<0.001	1.07 (1.05 ~ 1.09)
White cell count	0.09	0.02	5.01	<0.001	1.10 (1.06 ~ 1.14)

β, partial regression coefficient of variables in logistic regression; S.E, standard error; OR, odd ratio; CI, confidence interval; BMI, body mass index.

### 3.4 Evaluation of discrimination and calibration of the multivariate logistic prediction model for risk of hyperlipidemia

**Figure 3:**
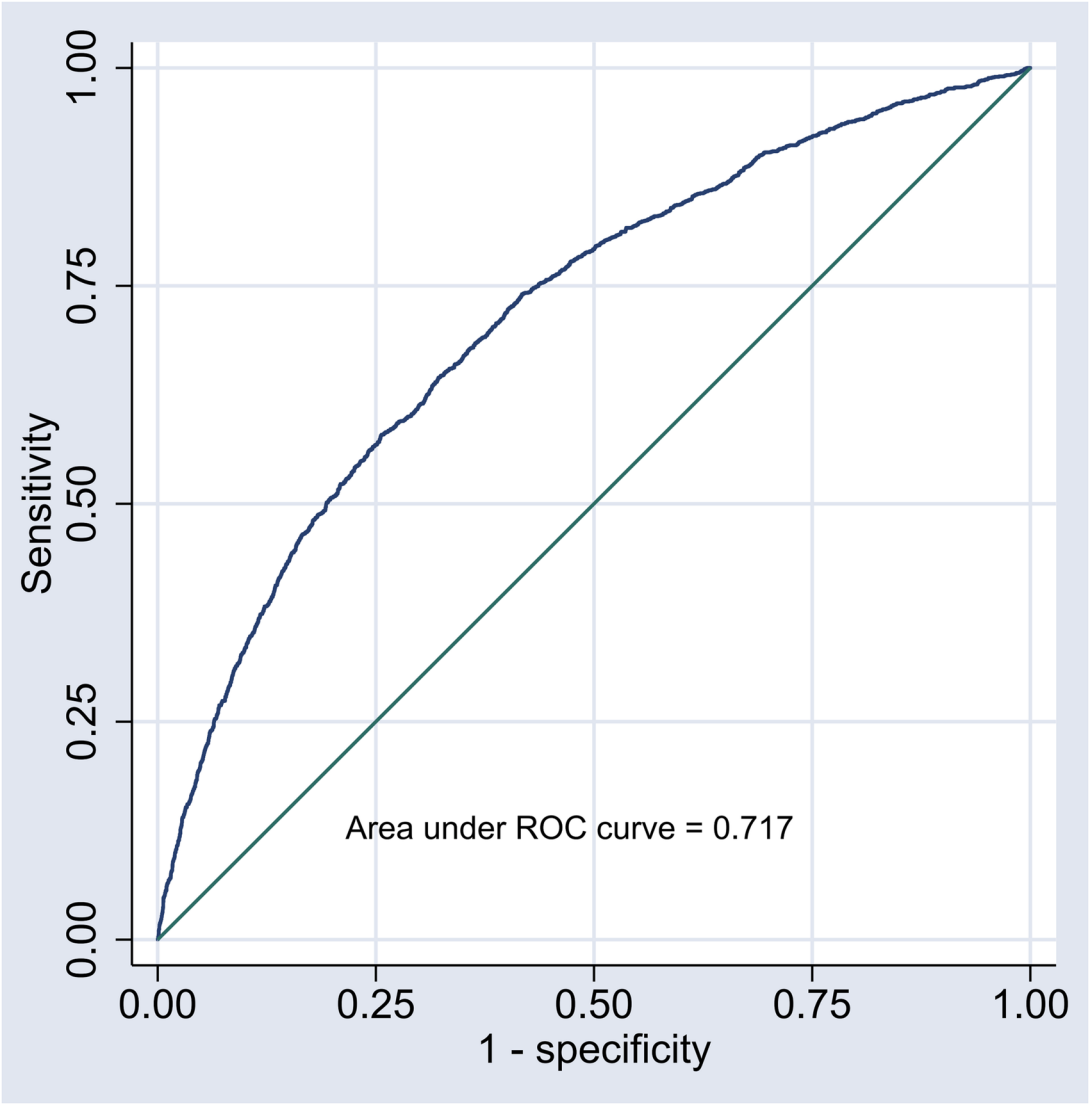
ROC curve of the nomogram model.

The discrimination of the logistic model was evaluated using the area under the ROC curve (AUC). The AUC for the logistic model in predicting the risk of hyperlipidemia in the middle-aged and older adult individuals was 0.717 (95% Confidence Interval (CI): 0.703–0.731), which indicates that the logistic model has good discrimination. For a visual representation, refer to Figure 3.

**Figure 4:**
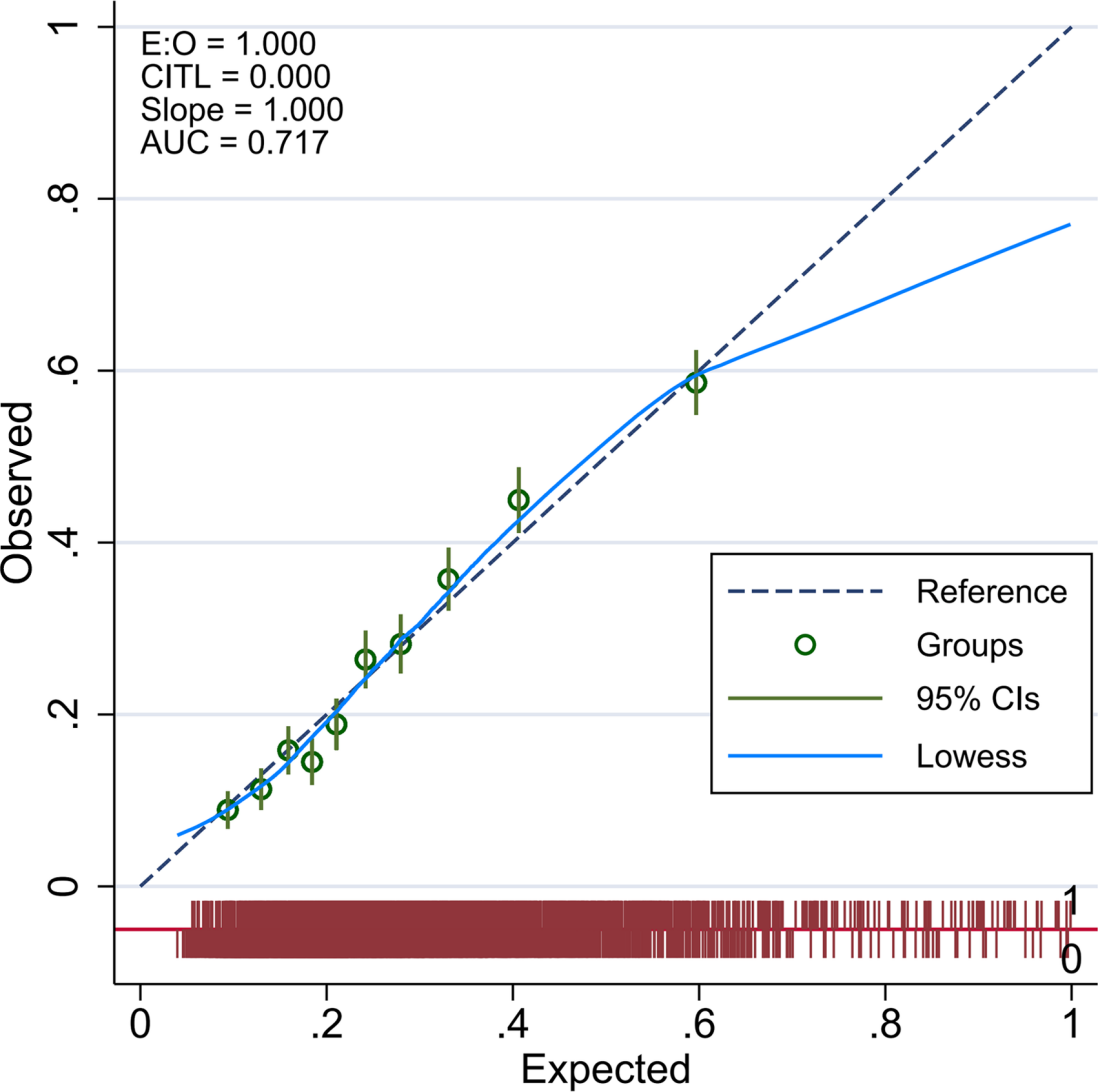
Calibration curve of the nomogram model.

The calibration of the logistic model was assessed using the Spiegelhalter’s z-statistic test, the calibration curve, and the Brier score. The *p*-value of the Spiegelhalter’s z-statistic test was 0.560, suggesting that the predicted probabilities align well with the actual frequencies of hyperlipidemia. Additionally, the Brier score for the nomogram model was 17.1%, which is below the threshold of 25%, indicating a good calibration degree. The calibration curve, as shown in Figure 4, also demonstrates that the nomogram model’s fit curve is consistent with the ideal curve.

To ensure the model’s robustness and to prevent over-fitting, Bootstrap self-sampling was performed for 500 iterations as an internal validation technique. Post-verification, the C-statistic for the nomogram model was 0.716, and the Brier score was 11.4%, suggesting that the model maintains good prediction efficiency and stability.

### 3.5 Evaluation of the clinical applicability of the nomogram prediction model for the risk of hyperlipidemia

**Figure 5:**
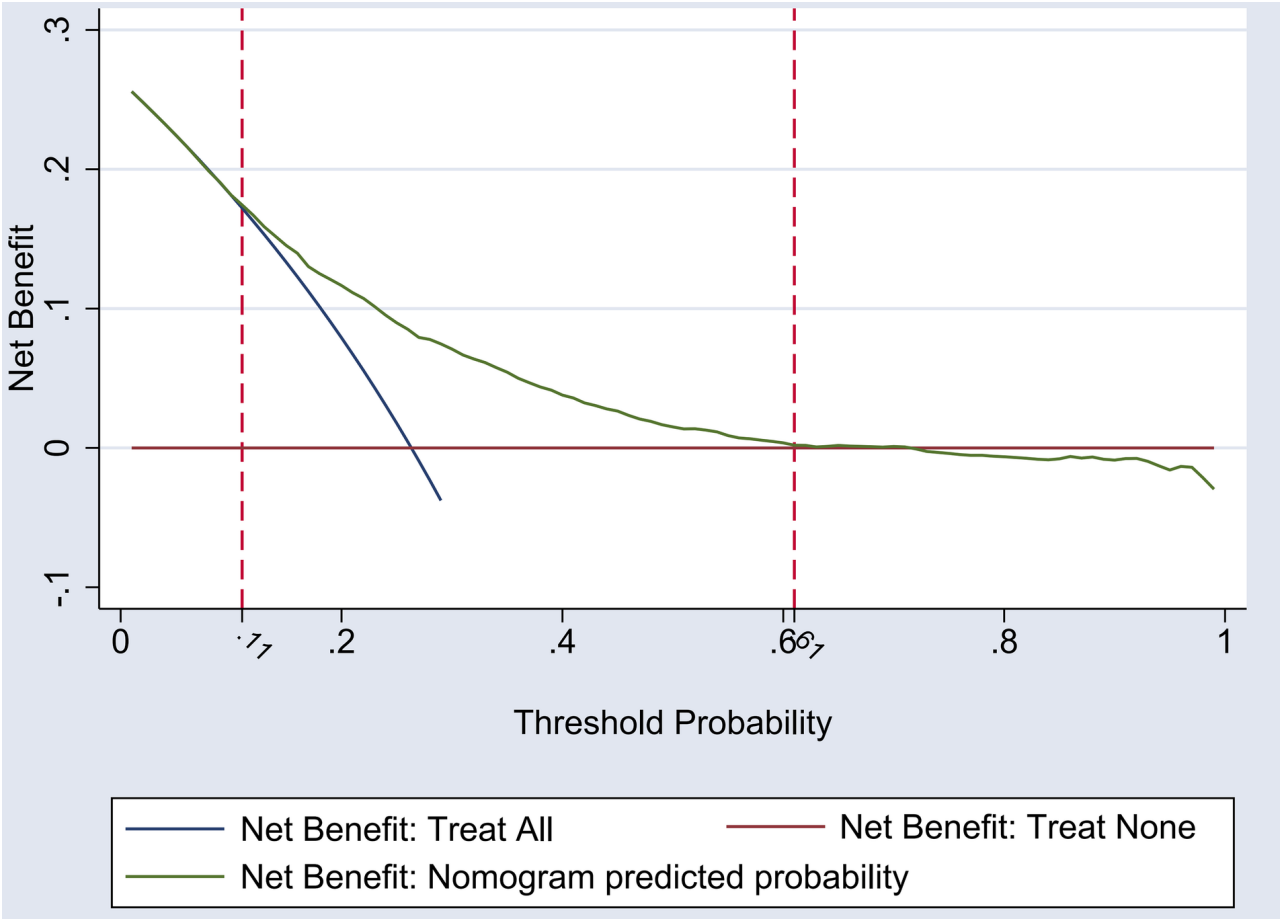
DCA curve of nomogram model for risk of hyperlipidemia.

The Decision Curve Analysis (DCA) curve was employed to assess the clinical applicability of the nomogram model for predicting hyperlipidemia risk in the middle-aged and older adult population. The net benefit in the DCA curve was defined as the difference between the total benefits and total costs associated with a particular intervention or treatment strategy. It was calculated by summing the individual benefits (such as improved health outcomes or cost savings) and subtracting the individual costs (including intervention costs and potential adverse effects), with each component weighted according to its respective probability and value. This calculation was performed at various threshold probabilities, and the resulting net benefits were plotted to form the DCA curve, providing a visual representation of the intervention’s cost-effectiveness across different decision-making thresholds. The findings indicate that when the predicted probability of hyperlipidemia lies within the range of 0.11 to 0.61, the use of the nomogram yields the highest net benefit for this demographic. This suggests that the nomogram model possesses favorable clinical utility. For a visual representation, refer to Figure 5.

### 3.6 The construction of nomogram prediction model for the risk of hyperlipidemia in middle-aged and older adult individuals

**Figure 6:**
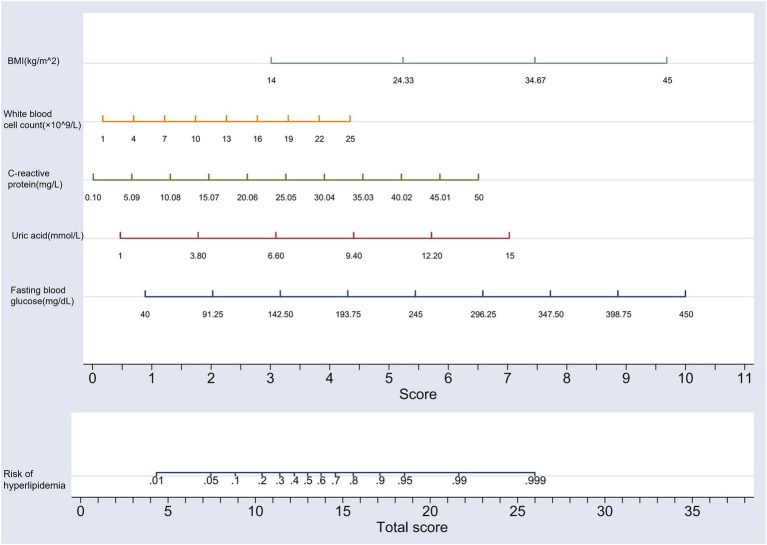
Nomogram prediction model of hyperlipidemia risk in middle-aged and older adult individuals.

Based on the five independent risk factors identified in Table 2, a nomogram model was developed as a predictive tool. The nomogram procedure involved the creation of a graphical tool that translates statistical prediction models into a user-friendly, visual format for clinical decision-making. This was achieved by mapping the logarithm of the odds of an event onto a scale, where each predictor variable’s contribution to the log-odds was represented by a series of parallel lines or axes. The user would then locate the value of each predictor on its corresponding axis and draw lines to a common scale that summed these values, providing a total score that could be translated back into a probability of the event of interest using a calibration curve or lookup table. The construction of the nomogram was facilitated using the statistical software Stata 17.0, with the final model depicted in Figure 6.

## 4 Discussion

According to the China Cardiovascular Health and Disease Report 2022 (17), there is a concerning trend of increasing prevalence in cardiovascular diseases (CVD) within China. The report estimates a staggering 330 million individuals are affected by CVD in China. This includes a significant number of patients with various heart conditions such as 11.39 million with coronary heart disease, 8.9 million with heart failure, 2.5 million with rheumatic heart disease, 2 million with congenital heart disease, and 245 million with hypertension. CVD hold the distinction of being the leading cause of death among both urban and rural residents in China. It is a stark reality that two out of every five deaths are attributable to CVD. Hyperlipidemia stands as one of the primary culprits in the development of atherosclerosis and its related diseases. This condition is not merely detrimental to health but also characterized by a high prevalence, representing a significant and widespread public health challenge (18). Due to the typically asymptomatic nature of hyperlipidemia, it often goes unnoticed, leading to a missed opportunity for the most effective prevention and treatment. Hence, evaluating the risk of hyperlipidemia is crucial for its timely detection and prevention. Analyzing data from the 2015 China Health and Retirement Longitudinal Study (CHARLS), The study identified a hyperlipidemia prevalence rate of 26.32% among China’s middle-aged and older adult population, a figure that, while slightly lower than the 38.04% reported by Liang Xiaoli and colleagues in a single-center community study (19), still indicates a significantly high prevalence of hyperlipidemia within this demographic. This underscores the importance of developing a predictive model that incorporates the risk factors specific to middle-aged and older adult individuals for the forecasting of hyperlipidemia risk.

LASSO regression analysis and Multivariate Logistic regression analysis has pinpointed several independent risk factors for hyperlipidemia within the middle-aged and older adult population. These factors include Body Mass Index (BMI), fasting blood glucose, serum uric acid, C-reactive protein, and white blood cell count. Each of these factors demonstrated an odds ratio (OR) greater than 1, and the *p*-values were found to be less than 0.05. A study conducted by Liu Wei and colleagues has identified BMI as an independent influencing factor in the development of hyperlipidemia. The study reported an odds ratio (OR) of 1.489 for BMI, with a p-value of 0.025, denoting a positive correlation between higher BMI and the presence of hyperlipidemia (20). The findings from this study are in alignment with those of Liu Wei et al. Hyperlipidemia is characterized by an elevated level of triglycerides or cholesterol in the blood. As BMI increases, it can result in the accumulation of adipose tissue, which in turn can elevate blood lipid levels, disrupt lipid metabolism, and subsequently heighten the risk of hyperlipidemia. Moreover, an elevation in BMI is not only a marker for increased adiposity but also interlinked with other comorbidities. Conditions such as insulin resistance and an enhanced inflammatory response are exacerbated by a higher BMI and are recognized contributors to the exacerbation of hyperlipidemia risk.

In the demographic of middle-aged and older adult individuals, the ascendance of fasting blood glucose levels is often indicative of a waning insulin secretory capacity coupled with an increased insulin resistance (21). Insulin plays a crucial role in the regulation of blood sugar levels. When there is insulin resistance, the cells are less responsive to the hormone, leading to ineffective utilization of glucose. This results in an increase in blood glucose concentration. The elevated blood sugar levels then act as a stimulus for the liver to synthesize additional triglycerides. Furthermore, insulin resistance is associated with a reduction in high-density lipoprotein (HDL) cholesterol levels, which is often termed the ‘good cholesterol’ due to its protective role against cardiovascular diseases. The combination of increased triglycerides and decreased HDL cholesterol contributes to an elevated risk of hyperlipidemia (22). The renal excretion of uric acid involves a complex interplay between filtration and reabsorption, a process that can be influenced by myriad factors, including dysregulated lipid metabolism. When there is a perturbation in lipid metabolism, it can have a deleterious effect on renal function, potentially leading to a diminished excretion of uric acid. This reduction in uric acid clearance can result in an elevation of blood uric acid levels, a condition known as hyperuricemia. Furthermore, hyperuricemia can engage in a vicious cycle by exacerbating lipid metabolic abnormalities through the mechanisms of oxidative stress and inflammatory responses. Consequently, an increase in serum uric acid levels serves not merely as an indicator of hyperlipidemia but also acts as a catalyst in its progression (23). An elevation of C-reactive protein (CRP) is a biomarker that reflects an increase in inflammatory activity within the body. Among middle-aged and older adult individuals, such an increase in inflammation is intimately linked to the onset and progression of atherosclerosis. Atherosclerosis is a complex disease characterized by inflammation of the blood vessel walls and the deposition of lipids, marking it as a significant risk factor for cardiovascular disease. Moreover, the inflammatory process can also disrupt blood lipid metabolism, thereby amplifying the risk of hyperlipidemia (24). In the middle-aged and older adult population, an increase in white blood cell (WBC) count is often associated with conditions such as chronic inflammation and infection. Chronic inflammation is recognized as a significant risk factor for a range of diseases, including cardiovascular diseases and diabetes, which in turn can elevate the risk of hyperlipidemia. Furthermore, a heightened WBC count can lead to impairment of vascular endothelial cell function, a factor that can exacerbate the risk of hyperlipidemia (25).

A nomogram is a graphical predictive tool designed to quantify the probability of a specific event in a user-friendly and intuitive manner. Typically, this process involves assigning scores to various variables, cumulating these scores to obtain a total score, and then utilizing this score to estimate the likelihood of the event in question. The nomogram’s operation is straightforward, and its visual representation of probabilities offers a high level of clarity, making it a practical and accessible method for risk assessment (26). Research has demonstrated the utility of the nomogram model in predicting a range of outcomes specifically in the middle-aged and older adult population. It has been effectively used to predict the risk of mild cognitive impairment (27), the risk of distant metastasis of chondrosarcoma (28), the risk of all-cause mortality (29), and the risk of multimorbidity diseases (30). These applications underscore the nomogram’s versatility and its effective performance in a spectrum of predictive tasks concerning health risks within the middle-aged and older adult demographic. However, to date, there exists a gap in representative research on nomogram prediction models specifically tailored to estimate the risk of hyperlipidemia in this age group. Addressing this research void, the current study endeavors to construct a nomogram prediction model aimed at forecasting hyperlipidemia risk among middle-aged and older adult individuals. This model is developed through an analytical examination of the survey data sourced from the 2015 China Health and Retirement Longitudinal Study (CHARLS) database. The nomogram model’s efficacy is substantiated through a battery of validation techniques including the AUC, DCA, the Spiegelhalter’s z-statistic test, and Bootstrap self-sampling internal verification. These assessments collectively confirm the model’s favorable predictive efficiency, clinical applicability, and stability. By integrating multiple risk factors, the nomogram provides a comprehensive assessment of hyperlipidemia risk for the middle-aged and older adult, facilitating individualized intervention strategies and enabling a more optimized allocation of medical resources. This approach is instrumental in enhancing the efficiency of disease prevention and management protocols.

### 4.1 Limitations

There are several limitations to be acknowledged in the current study. Firstly, the research population is derived from middle-aged and older adult participants of the CHARLS, which may limit the generalizability of the prediction model to younger demographics, children, and individuals with varying socio-economic backgrounds, cultural practices, genetic profiles, or health statuses. Secondly, despite the representative nature of the study, the sample size post-screening is constrained. While internal validation of the nomogram model was performed using Bootstrap resampling, the sample size limitation precluded external validation. Therefore, the need remains for a prospective, multi-center, and large-sample cohort study to confirm the extrapolation and stability of the model developed in this research. Lastly, the scope of research indicators included in this study is limited. Future studies could benefit from incorporating a broader array of predictive indicators associated with hyperlipidemia to enhance the model’s predictive accuracy and to further refine its efficiency.

## 5 Conclusion

The prevalence of hyperlipidemia among China’s middle-aged and older adult population is determined to be 26.32%. Through rigorous analysis, BMI (Body Mass Index), fasting blood glucose, serum uric acid, C-reactive protein, and white blood cell count have been established as independent risk factors for the development of hyperlipidemia in this age group. The nomogram model developed for assessing hyperlipidemia risk in the middle-aged and older adult demonstrates excellent predictive efficiency and clinical applicability. It serves as a valuable tool to aid community health workers and medical professionals in the identification of high-risk hyperlipidemia groups. By employing this model, targeted preventive strategies can be implemented, which may lead to a reduction in the incidence of hyperlipidemia among the middle-aged and older adult demographic.

## Data Availability

The datasets presented in this study can be found in online repositories. The names of the repository/repositories and accession number(s) can be found at: the data used in this study is from CHARLS (2015). Data are publicly available and can be downloaded from CHARLS website: https://charls.pku.edu.cn/.
